# Intracranial Aneurysm Formation in Type-One Diabetes Rats

**DOI:** 10.1371/journal.pone.0067949

**Published:** 2013-07-02

**Authors:** Tao Yan, Michael Chopp, Ruizhuo Ning, Alex Zacharek, Cynthia Roberts, Jieli Chen

**Affiliations:** 1 Department of Neurology, Tianjin Neurological Institute, Tianjin Medical University General Hospital, Tianjin, China; 2 Department of Neurology, Henry Ford Hospital, Detroit, Michigan, United States of America; 3 Department of Physics, Oakland University, Rochester, Michigan, United States of America; University of Pécs Medical School, Hungary

## Abstract

**Background & Objective:**

Diabetes mellitus (DM) plays an important role in the pathogenesis of vascular complications including arteriosclerosis and ischemic stroke. Whether DM impacts intracranial aneurysm (IA) formation has not been extensively investigated. In this study, we tested the underlying mechanism of type one DM (T1DM) induced IA formation in rats.

**Experimental Approaches:**

T1DM was induced by streptozotocin injection. Rats were euthanized at 0, 4 and 10 weeks after T1DM induction. To evaluate cerebral vascular perfusion, Fluorescein isothiocyanate - dye was injected at 5 min prior to euthanasia. Vascular perfusion was measured by laser scanning confocal microscopy. Trichrome, Elastica van Gieson, alpha-smooth muscle actin (a-SMA) and receptor of advanced glycation end-products (RAGE), toll-like receptor 4 (TLR4) and matrix metalloproteinase 9 (MMP9) immunostaining were performed. The IA formation was classified by 0–3 stages: 0: Normal; 1: Endothelial damage; 2: Moderate protrusion; and 3: Saccular aneurysm formation.

**Results:**

T1DM significantly increased IA formation identified by the classification of aneurysmal changes compared with non-DM rats (p<0.05). However, T1DM induced IA formations were classified as stage 1 and stage 2, but not stage 3. Cerebral vascular perfusion was significantly decreased in T1DM rats compared to non-DM rats (p<0.01). DM10W rats exhibited a significant decrease of cerebral vascular perfusion compared to DM4W rats (p<0.05). T1DM rats also significantly increased the internal carotid artery (ICA) intimae and media thickness, and decreased the internal carotid artery diameter compared to non-DM rats. RAGE, MMP9 and TLR4 expression were significantly increased in T1DM rats compared to non-DM rats. The increased RAGE, TLR4 and MMP9 significantly correlated with IA formation (p<0.05).

**Conclusion:**

T1DM increases IA formation. The increased RAGE, MMP9 and TLR4 expressions might contribute to IA formation in T1DM rats.

## Introduction

Hyperglycemia and diabetes play an important role in the pathogenesis of vascular complications including macroangiopathy and microangiopathy [1], which lead to retinopathy, nephropathy, arteriosclerosis and increased ischemic stroke risk by 2–4 fold relative to those without diabetes [2]. Diabetes mellitus (DM) induces vascular endothelial damage and dysfunction, decreases cerebral tight junction protein expression [3], and promotes artery intima-media thickness (IMT) [4] and atherosclerotic vascular disease. Endothelial damage [5] and reduction of tight junction protein expression are also related with cerebral aneurysm formation [6]. However, DM has been related to a decreased risk of aneurysm rupture in patients 60 years or older and in women [7] and does not predispose to the development or rupture of saccular cerebral aneurysms [8], [9], [10]. Patients with aneurysmal subarachnoid hemorrhages have a lower or equivalent prevalence of DM than the general population [8], [9], [10]. The mechanisms responsible for this negative association remain unknown.

Atherosclerotic blood vessels in diabetes are associated with inflammation and remodeling of the extracellular matrix. Advanced glycation end-products (AGEs) are a complex group of compounds formed via a nonenzymatic reaction between reducing sugars and amine residues on proteins, lipids, or nucleic acids. Receptor of advanced glycation end-products (RAGE) is the receptor of AGEs [11], [12]. The AGE/RAGE signaling pathway plays a critical role in arterial diseases that are characterized by endothelial dysfunction, accumulation of extracellular matrix proteins, intima-media thickening, and decreased elasticity, which accelerate the development of atherosclerosis in the diabetic patients and animals [13]. RAGE also promotes the development of abdominal aortic aneurysms by inducing matrix metalloproteinase 9 (MMP9) expression [14]. MMP9 degrades the extracellular matrix and is involved in control and regulation of inflammation [15]. Increased MMP9 expression was detected in stenotic and aneurysmal arterial remodeling [16]. As a proinflammatory factor, toll-like receptor 4 (TLR4) upregulates MMP9 expression [17] and mediates inflammatory responses and also contributes to arteriosclerosis [18], [19]. Our previous study has found that T1DM-MCAo rats exhibit significantly increased RAGE, TLR4 and MMP9 expression in macrophages in the ischemic brain compared to the ischemic brain of wild-type (WT) rats [20].

Generally, smoking, excessive alcohol, untreated hypertension and female gender have been shown to be the most important risk factors for aneurysmal subarachnoid hemorrhage (SAH) [8], [21]. Recent, large-scale genome-wide association (GWA) studies have revealed consistent and replicable genetic markers of several complex diseases such as coronary artery disease, and type 2 diabetes may also contribute to IA development [22]. In this study, we investigated the effect of T1DM on IA formation and the underlying mechanism by which T1DM induces IA formation in rats.

## Materials and Methods

All experimental procedures were carried out in accordance with the NIH Guide for the Care and Use of Laboratory Animals and approved by the Institutional Animal Care and Use Committee of Henry Ford Hospital (IACUC approval number: 999). All efforts were made to ameliorate suffering of animals.

### Diabetes Induction

Adult Male Wistar rats (250–275 g) purchased from Charles River (Wilmington, MA) were used. Diabetes was induced by a single intraperitoneal injection of streptozotocin into rats (STZ, 60 mg/kg, dissolved in citrate buffer, pH 4.5; Sigma Chemical Co., St. Louis, MO). The fasting blood glucose level was measured 10 days after STZ injection by using a glucose analyzer (Accu-Chek Compact System; Roche Diagnostics, Indianapolis, IN) with test strips for glucose (Polymer Technology System, Inc. Indianapolis, IN) according to the manufacturer’s instructions. Diabetes was defined by a fasting blood glucose exceeding 300 mg/dl. All animals included in this study also had a fasting blood glucose exceeding 300 mg/dl ten days after streptozotocin injection and at euthanasia.

### Experiment Groups

Wild type (WT) non-diabetic rats were used as control (n = 7). T1DM rats were euthanized 4 weeks (DM4W, n = 7) or 10 weeks (DM10W, n = 7) after STZ injection. Immunostaining was performed on all rats.

### Histological and Immunohistochemical Assessment

The brains were fixed by transcardial perfusion with saline, followed by perfusion and immersion in 4% paraformaldehyde before being embedded in paraffin. For immunostaining, a standard paraffin block was obtained from the bregma (−1 mm to 1 mm) of the brain. A series of 6 µm thick sections were cut from the block. Every 10th coronal section for a total of 5 sections was used for immunohistochemical staining. Immunostaining for Trichrome (for differentiating muscle from collagen of arteries), α-smooth muscle actin (α-SMA, a smooth muscle cell marker, mouse monoclonal IgG 1∶800, Dako) and inflammatory mediators including RAGE (1∶400; Dako, Carpinteria, CA, USA), MMP9 (1∶500, Santa Cruz Biotechnology, Santa Cruz, CA, USA) and TLR4 (goat polyclonal IgG; dilution 1∶100; Cruz Biotech Inc., Santa Cruz, California) immunostaining were performed. Elastica van Gieson staining was used to show the thinning and loss of elastic tissue fibers in aneurysmal formation [23]. Control experiments consisted of staining brain coronal tissue sections as outlined above, but non-immune serum was substituted for the primary antibody. The immunostaining analysis was performed by an investigator blinded to the experimental groups.

### Classification of Aneurysmal Changes

Classification of aneurysmal changes at the anterior cerebral artery-olfactory artery branching sites using microscopy findings with Elastica van Gieson staining were recorded as: (A) Normal (stage 0). (B) Endothelial damage (stage 1). (C) Moderate protrusion (stage 2). (D) Saccular aneurysm (stage 3) [23], [24], [25].

### Quantification of RAGE, MMP9 and TLR4 Expression

For quantitative measurements of RAGE, MMP9 and TLR4, five slides from each brain, with each slide anterior cerebral artery and olfactory artery were digitized under a 20× objective (Olympus BX40) using a 3-CCD color video camera (Sony DXC-970MD) interfaced with an MCID image analysis system (Imaging Research, St. Catharines, Canada) [26], [27]. RAGE, MMP9 and TLR4 were measured and expressed as ratio of positive area to luminal area of the anterior cerebral artery and olfactory artery. Data were analyzed in a blinded manner.

### Trichrome Immunostaining and Measurement

Using Gomori One-Step Trichrome Stain (Sigma, St Louis, MO), brain sections were postfixed in Bouin fixative. Nuclei are stained with Weigert hematoxylin and then stained in Gomori trichrome stain followed by a 0.5% acetic water rinse. Connective tissue and collagen are stained blue, nuclei are stained dark red/purple, and cytoplasm is stained red/pink. Artery intimae, media, and artery diameter (minimum diameter) were measured in the internal carotid artery (ICA).

### 251658240αSMA-positive Coated Arterial Diameter and Wall Thickness

The α-SMA stained vessels were analyzed with regard to small and large vessels (≥10 µm diameter). The 10 largest arterial wall thicknesses and internal arterial diameters were measured. In addition, the total number of occluded arterioles in the bilateral hemispheres was counted.

### Double Immunohistochemical Staining

To specifically identify RAGE, TLR4 and MMP9-reactive cells co-localized with macrophages (ED1), double immunofluorescence staining of RAGE/ED1, TLR4/ED1 and MMP9/ED1 were performed. Fluorescein isothiocyanate (FITC, Calbiochem, San Diego, CA, USA), 4, 6-diamidino-2-phenylindole (DAPI, Vector Laboratories) and cyanine-3 (CY3, Jackson Immunoresearch, PA, USA) were used for double-label immunoreactivity. Each coronal section was first treated with the primary anti-RAGE, anti-TLR4 or anti-MMP9 antibody with Cy3, and then followed by ED1 with FITC. Control experiments consisted of staining brain coronal tissue sections as outlined above, but using nonimmune serum for the primary antibody.

To test cerebral vascular perfusion, FITC-dye (50 mg/rat in 2 ml PBS, IV) was injected at 5 min before euthanasia in another group of animals (n = 4/group). Animals were anesthetized with ketamine and fixed by 4% paraformaldehyde. The brain tissues were processed to acquire adjacent 100-µm thick coronal sections using a vibratome. Five sections from the bregma (−1 mm to 1 mm) section were used to detect vascular perfusion by laser scanning confocal microscopy (LSCM) with the use of a Bio-Rad MRC 1024 (argon and krypton) laser-scanning confocal imaging system mounted onto a Zeiss microscope (BioRad) [28]. For FITC labeled coronal sections, green (FITC) fluorochromes on the sections were excited by a laser beam at 488 nm; emissions were sequentially acquired through 522 nm emission filters. Areas of interest were scanned with an ×20 objective lens in 512.2×512.2-µm format in the x-y direction and 0.5 µm in the z direction. The photos and quantification of FITC labeled vessels were processed by ImageJ (Wayne Rasband, National Institutes of Health, USA).

### Statistical Analysis

All measurements and analyses were performed by normality of distribution, and the homogeneity of variances was tested including the biochemistry, and immunostaining. One-way analysis of variance (ANOVA) was used for the immunostaining analysis. Spearman correlation coefficients were calculated to study the correlation between aneurysm formation and immunohistochemical measurements. All data are presented as mean ± standard error (SE).

## Results

### T1DM Increases Intracranial Aneurysm (IA) Formation (Figure 1)

**Figure 1:**
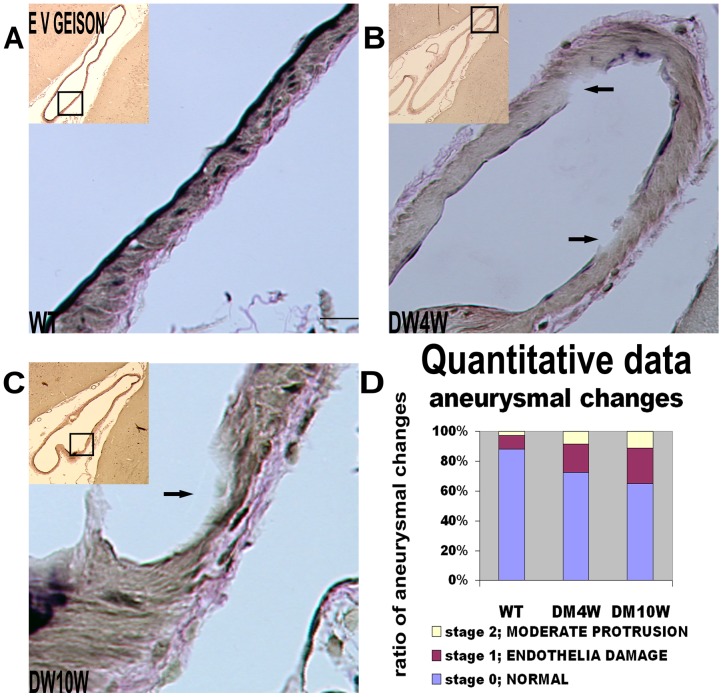
T1DM increases IA formation identified by the classification of aneurysmal changes compared with non-DM rats. **A–C:** Elastica Van Geison staining: T1DM increases IA formation compared with non-DM rats. **D:** Quantitative data: The ratio of stage 1 and stage 2 of aneurysmal formations to total arterial number was significantly increased in T1DM rats (p<0.05). Arrows show a slight focal thinning and bulging of the arterial wall (Fig. 1B and C). Scale bar in A, 20 µm.

To test whether diabetes regulates IA formation and alters elastic tissue fibers in the anterior cerebral artery-olfactory artery branching sites, Elastica van Gieson staining was performed [29]. We found that T1DM significantly increased aneurysmal formation identified by the classification of aneurysmal changes compared with non-DM rats (Figure 1A–C). The ratio of stage 1 and 2 aneurysmal formations to total artery number increased significantly in T1DM rats compared to non-DM rats (Figure 1D, *p<*0.05), and all of the aneurysmal formations were in stage 1 and stage 2, but not in stage 3 (Figure 1D).

### T1DM Increases RAGE, MMP9 and TLR4 Expression (Figure 2)

**Figure 2:**
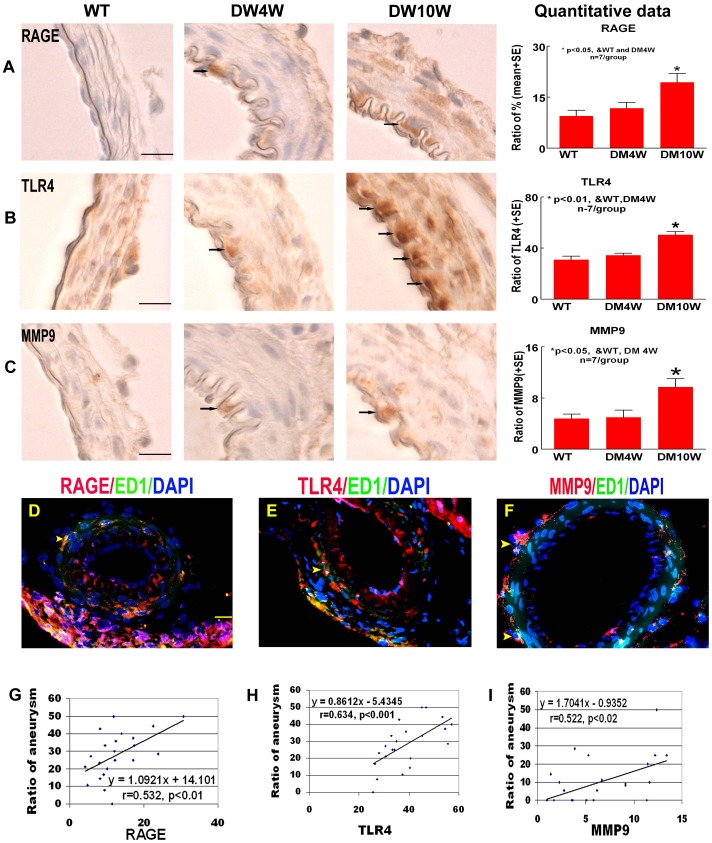
T1DM increases RAGE, MMP9 and TLR4 expression. RAGE, TLR4 and MMP9 is correlated with aneurysm formation in T1DM rats. **A–C**: RAGE (A), TLR4 (B) and MMP9 (C) immunostaining and quantitative data. T1DM significantly increased RAGE, MMP9 and TLR4 expression compared to non-DM rats. Arrows indicate the positive cells in the arterial wall. **D–F:** Double immunostaining ED1 with RAGE (D), TLR4 (E) and MMP9 (F). MMP9, TLR4 and RAGE expression is colocalized with ED1. Arrow heads indicate the positive cells in the arterial wall. **G–I:** Correlation analysis of aneurysm formation with RAGE (G), TLR4 (H) and MMP9 (I). Aneurysm formation significantly correlated with RAGE, TLR4 and MMP9 expression. Scale bar in A, D, 20 µm.

To obtain insight into the possible underlying mechanisms of T1DM-induced arthrosclerosis and aneurysmal formation, RAGE, MMP9 and TLR4 expressions were measured. Figure 2 shows that T1DM significantly increased RAGE, MMP9 and TLR4 expression compared to non-DM rats at 10 weeks after diabetes induction (*p<*0.05, Figure 2A–C). Figure 2D–F show that RAGE, TLR4 and MMP9 are predominantly detected in macrophages (ED1 positive cells) in the cerebral arteries. The results are consistent with previous studies [14], [20]. In addition, Figure 2G–I show that the increase of RAGE (r = 0.532, *p<*0.01; Figure 2G), TLR4 (r = 0.634, *p<*0.001; Figure 2H) and MMP9 (r = 0.522, *p<*0.02; Figure 2I) expression significantly correlated with IA formation in T1DM rats.

### T1DM Increases the ICA Intimae Thickness and Media Thickness, and Decreases the ICA Internal Diameter in Trichrome Staining (Figure 3)

**Figure 3:**
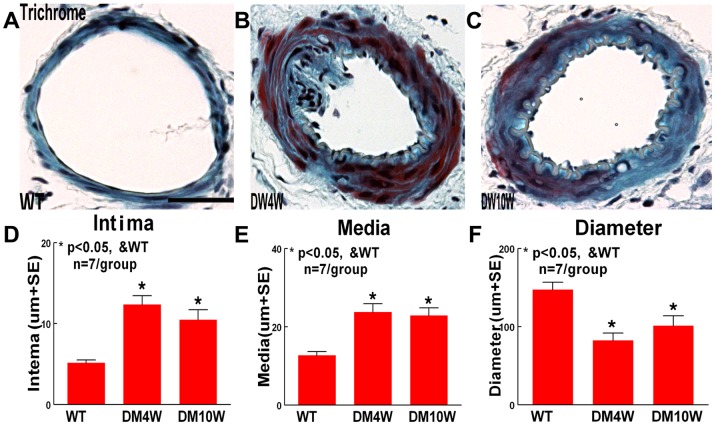
T1DM accelerates arteriosclerosis-like changes in ICA. A–C: Trichrome staining in WT (A), 4 week T1DM (B) and 10 week T1DM (C) rats. D–F: Quantitative data. T1DM increases the ICA intimae (D) and media thickness (E), and decreased the ICA diameter (F) (p<0.05). Scale bar in A, 0.1 mm.

To test why T1DM increases IA formation in stage 1 and 2, but not in stage 3, and whether T1DM induces cerebral arteriosclerosis-like changes, Trichrome staining was used. Figure 3 shows that ICA intimae thickness and media thickness were significantly increased in T1DM rats compared with non-DM rats (p<0.05, Figure 3D, E). The ICA internal diameter was significantly decreased in T1DM rats compared with non-DM rats (*p<*0.05, Figure 3F).

### T1DM Decreases Cerebral Vascular Perfusion (Figure 4)

**Figure 4:**
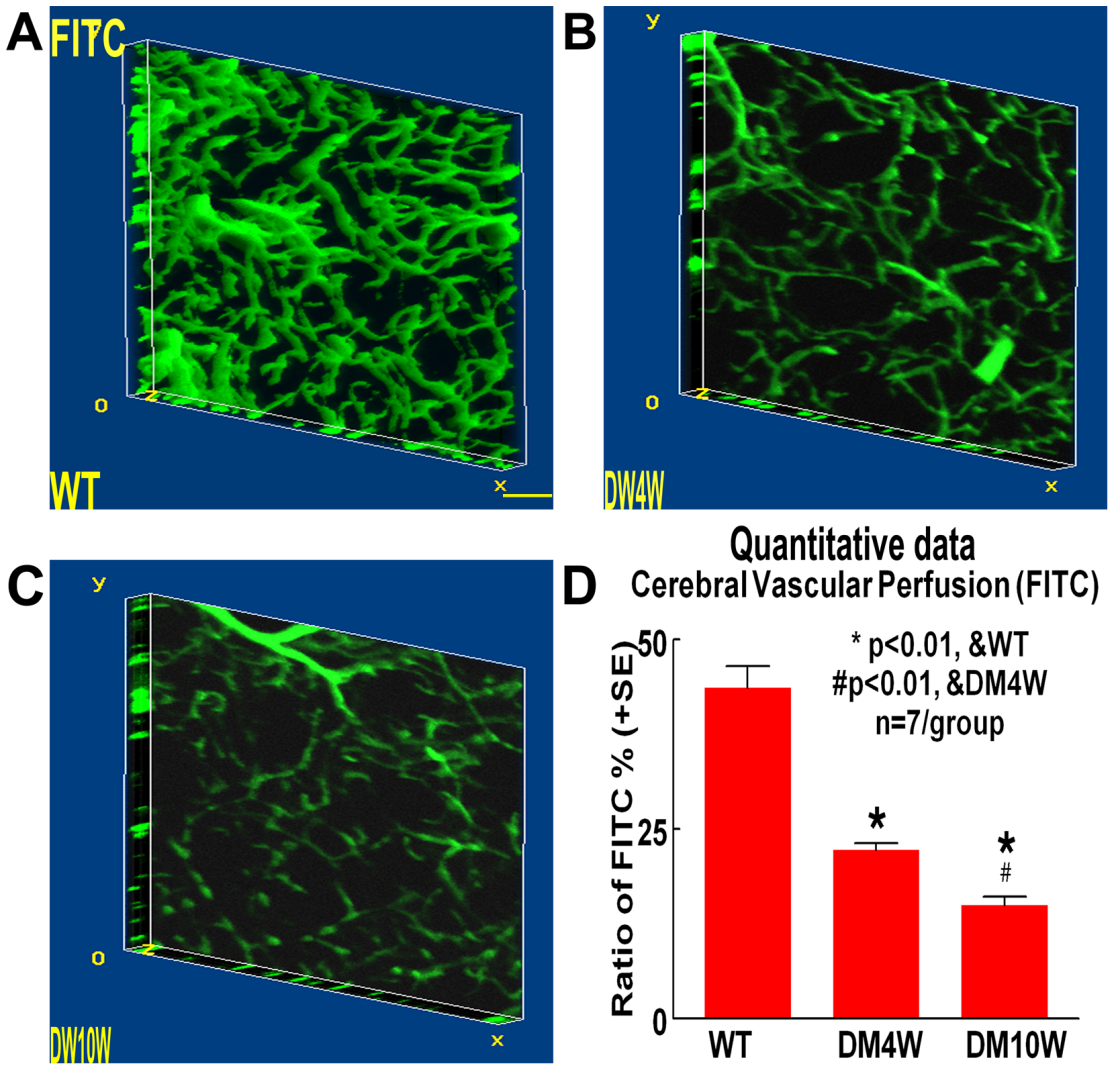
T1DM decreases cerebral vascular perfusion in the cerebral parenchyma compared to non-DM rats. A–C: FITC-dye vascular perfusion in WT (A), 4 weeks T1DM (B) and 10 weeks T1DM (C) rats. D: Cerebral vascular perfusion quantitative data. Scale bar in A, 0.1 mm.

To test whether T1DM regulates cerebral vascular perfusion, FITC-dye was injected into rats. Figure 4 shows that the cerebral vascular perfusion significantly decreased at both time points (DM4W and DM10W) in T1DM rats compared to non-DM rats (*p<*0.01, Figure 4D), and the cerebral vascular perfusion of DM10W was more attenuated compared to DM4W(*p<*0.05, Figure 4D). The data indicate that cerebral perfusion is decreased with the course of diabetes.

### T1DM Decreases Cerebral Arterial Internal Diameter and Cerebral Vascular Perfusion, and Increases Cerebral Arterial Wall Thickness and Occluded Cerebral Arterioles Compared to Non-DM Rats (Figure 5)

**Figure 5:**
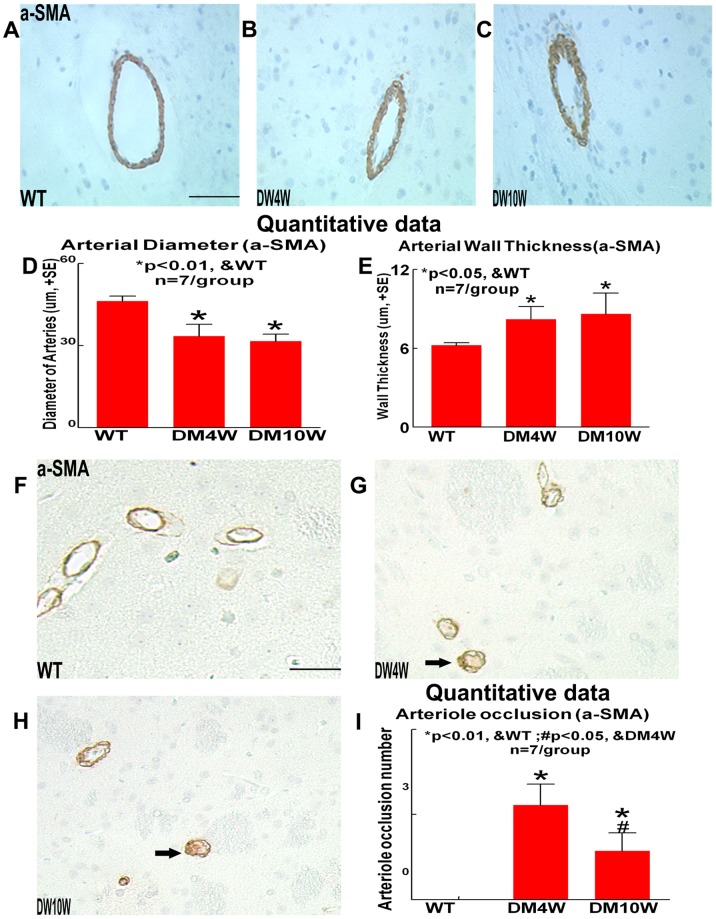
T1DM accelerates arteriosclerosis-like changes in cerebral arteries compared to non-DM rats. T1DM decreases the cerebral arterial diameter while increases the cerebral arterial wall thickness and arterioles occlusion in the cerebral parenchyma compared to non-DM rats. a-SMA immunostaining and quantitative data: A–C: Cerebral artery wall diameter and thickness in WT (A), 4 week T1DM (B) and 10 week T1DM (C) rats, D: quantitative data of arterial diameter, E: quantitative data of arterial wall thickness. F–H: Cerebral arterioles occlusion in WT (F), 4 week T1DM (G) and 10 week T1DM (H) rats. I: Cerebral arterioles occlusion quantitative data. Scale bar in A, F, 0.05 mm. Arrows indicate the occluded arterioles (G, H).

To test why T1DM decreases cerebral vascular perfusion, arterial internal diameter and occlusion cerebral arterioles number were measured. The cerebral arterial internal diameters were significantly decreased in T1DM rats at 4 weeks (DM4W) and 10 weeks (DM10W) after T1DM induction compared to non-DM rats (*p<*0.05, Figure 5D). Concomitantly, the cerebral artery wall thickness and numbers of occluded cerebral arterioles were significantly increased in DM4W and DM10W rats compared to non-DM rats (*p<*0.05, Figure 5E, I). In addition, there was no significant difference between DM4W and DM10W in arterial internal diameter and wall thickness (*p>*0.05, Figure 5D, E).

## Discussion

In this study, to our knowledge we are the first to demonstrate that T1DM promotes the formation of intracranial aneurysm as well as significantly increases RAGE, MMP9 and TLR4 expression in the intracranial arterial wall compared to WT non-DM rats. We also found that T1DM increases cerebral artery IMT and atherosclerosis-like changes identified by decreased arterial diameter and cerebral vascular perfusion, and significantly increases the arterial wall thickness compared to non-DM rats.

### Diabetes Increases Initial Stages of Intracranial Aneurysm Formation

Previous studies have found that diabetes increases vascular damage and atherosclerotic vascular disease [30], [31]. Consistent with these studies, we found that T1DM significantly increased artery IMT and vascular occlusion and decreased arterial diameter. There are several studies have investigated the effects of abdominal aortic aneurysm formation in diabetic population and demonstrated that diabetes does not aggravate aortic aneurysmal development [32], [33], [34]. However, T1DM significantly increased early stage intracranial aneurysmal formation (stage1 and 2), but not stage 3 intracranial aneurysmal formation. Our data suggest that T1DM promotes early intracranial aneurism formation, but does not promote aneurysm development to stage 3.

The reason for the reduced stage 3 aneurysm development in T1DM is not clear. Possible reasons may be related to: 1) dysregulation of tPA/PAI-1 signaling pathway. A previous clinical report showed that tPA thrombolysis could induce the rupture of cerebral aneurysms [35]. While hyperglycemia significantly increases PAI-1 expression [36] in cerebral arteries and also downregulates t-PA expression and activity [37], which may increase inflammatory cell accumulation in the lesioned vessels and increase arterial intima-media thickness, and thus attenuates aneurysm diameter [38], and thereby decreases saccular aneurysm (Stage 3 IA formation). 2) There may be vascular remodeling after arteriosclerosis in diabetics [16]. There are two major directions in which arterial remodeling may progress. Intima thickening and constrictive geometric remodeling of the artery wall are primary changes associated with the decreased lumen [16]. Expansive remodeling of the wall tends to preserve the lumen in the face of increased lesion burden. Therefore, the thicker intima-media and lower wall stress in diabetics may partly explain the protective effect of diabetes against aneurysm development [39]. 3) Hypertension is considered a risk for aneurysmal rupture [40], [41]. Previous studies have found that hypertension is more common in the diabetic population than in the general non-diabetic population [42], [43], and hypertension and/or insulin-dependent diabetes mellitus significantly increases cerebral IA formation [44], [45]. In the current study, we investigated the effects of T1DM alone on the regulation of IA formation. The effects of diabetes in combination with hypertension on the IA formation and progression warrants further investigation. In addition, tPA thrombolysis could induce rupture of cerebral aneurysms and also increase IA formation [32], [35]. While tPA treatment of ischemic stroke in T1DM stroke rats significantly increases brain hemorrhage formation [46], [47], whether the brain hemorrhage formation induced by tPA treatment is related with IA formation in T1DM animals, requires further investigation.

### Increase Levels of RAGE, TLR4 and MMP9 Might Promote the Initiation and Formation of Intracranial Aneurysm and Atherosclerosis-like Changes in T1DM Rats

AGEs accumulate in the vessel wall and are implicated in both the microvascular and macrovascular complications of diabetes [48]. The expression of the AGE receptor RAGE is upregulated in endothelial cells, smooth muscle cells, and mononuclear phagocytes in diabetic vasculature, and such upregulation is linked to the inflammatory response [49], [50], and it accelerates the development of atherosclerosis in patients with diabetes [13]. It has been generally accepted that the occurrence of aneurysm is related to the presence of severe atherosclerosis in the circulation [51]. Increased RAGE expression was detected in aneurysm formation in animal models and in human patients [14], [52]. RAGE affects the aneurysmal formation via nuclear factor kappa-light-chain-enhancer of activated B cells (NF-kB) pathway to activate MMP9 expression [8], [53]. In addition, TLR4 initiates inflammation in diabetics and plays an important role in arteriosclerosis by inducing inflammation responses [18], [19]. TLR4 expression is apparently upregulated in the endothelial cell layer and adventitia of aneurysm walls [54], and increases MMP9 expression in macrophages [27], [29], which promote aneurysmal formation [55], [56], [57]. MMP9 degrades especially type IV collagen, the main constituent of the basement membrane [58], and contributes to development of vascular lesions [59]. MMP9 is also involved in abdominal aortic aneurysm formation [32], [60], [61]. Inhibition of MMP9 therapy results in attenuation of aneurysm formation by suppression of inflammation of the aortic wall [62]. We found that diabetes significantly resulted in increased expression of RAGE, TLR4 and MMP9 in damaged arteries which also correlated with intracranial formation of aneurysms. The increased intracranial aneurysm formation may be regulated by inflammatory factors RAGE, MMP9 and TLR4 [63].

### Conclusions

In conclusion, this study demonstrates that Type-1 diabetes promotes cerebral aneurysmal formation as well as arteriosclerosis-like changes in T1DM rats. Inflammatory mediators including MMP9, RAGE and TLR4 in diabetes might contribute the increased initiation and formation of aneurysm and arteriosclerosis. This study provides mechanistic insight into how T1DM promotes initial development of intracranial aneurysm but limits further progression.

## References

[1] Li W, Prakash R, Kelly-Cobbs AI, Ogbi S, Kozak A, et al (2010) Adaptive cerebral neovascularization in a model of type 2 diabetes: relevance to focal cerebral ischemia. Diabetes 59: 228–235. 19808897 10.2337/db09-0902 PMC2797926

[2] Fox CS, Coady S, Sorlie PD, Levy D, Meigs JB, et al (2004) Trends in cardiovascular complications of diabetes. JAMA 292: 2495–2499. 15562129 10.1001/jama.292.20.2495

[3] Ye X, Chopp M, Cui X, Zacharek A, Cui Y, et al (2011) Niaspan enhances vascular remodeling after stroke in type 1 diabetic rats. Exp Neurol 232: 299–308. 21963653 10.1016/j.expneurol.2011.09.022 PMC3265018

[4] Haley AP, Gonzales MM, Tarumi T, Tanaka H (2012) Subclinical vascular disease and cerebral glutamate elevation in metabolic syndrome. Metab Brain Dis. 10.1007/s11011-012-9306-x PMC3614081 22552897

[5] Tamura T, Jamous MA, Kitazato KT, Yagi K, Tada Y, et al (2009) Endothelial damage due to impaired nitric oxide bioavailability triggers cerebral aneurysm formation in female rats. J Hypertens 27: 1284–1292. 19307983 10.1097/HJH.0b013e328329d1a7

[6] Tada Y, Yagi K, Kitazato KT, Tamura T, Kinouchi T, et al (2010) Reduction of endothelial tight junction proteins is related to cerebral aneurysm formation in rats. J Hypertens 28: 1883–1891. 20577123 10.1097/HJH;0b013e32833c2273

[7] Inagawa T (2010) Risk factors for the formation and rupture of intracranial saccular aneurysms in Shimane, Japan. World Neurosurg 73: 155–164; discussion e123. 10.1016/j.surneu.2009.03.007 20860953

[8] Feigin VL, Rinkel GJ, Lawes CM, Algra A, Bennett DA, et al (2005) Risk factors for subarachnoid hemorrhage: an updated systematic review of epidemiological studies. Stroke 36: 2773–2780. 16282541 10.1161/01.STR.0000190838.02954.e8

[9] Adams HP Jr, Putman SF, Kassell NF, Torner JC (1984) Prevalence of diabetes mellitus among patients with subarachnoid hemorrhage. Arch Neurol 41: 1033–1035. 6477209 10.1001/archneur.1984.04050210031009

[10] Greenhalgh RM, Laing S, Taylor GW (1980) Risk factors in carotid artery stenosis and intracranial aneurysms. J Cardiovasc Surg (Torino) 21: 559–567. 7451561

[11] Schmidt AM, Vianna M, Gerlach M, Brett J, Ryan J, et al (1992) Isolation and characterization of two binding proteins for advanced glycosylation end products from bovine lung which are present on the endothelial cell surface. J Biol Chem 267: 14987–14997. 1321822

[12] Neeper M, Schmidt AM, Brett J, Yan SD, Wang F, et al (1992) Cloning and expression of a cell surface receptor for advanced glycosylation end products of proteins. J Biol Chem 267: 14998–15004. 1378843

[13] Fujita T (2010) AGE/RAGE axis in the development of abdominal aortic aneurysm. Ann Surg 252: 203–205; author reply 205. 10.1097/SLA.0b013e3181e495e6 20562684

[14] Zhang F, Kent KC, Yamanouchi D, Zhang Y, Kato K, et al (2009) Anti-receptor for advanced glycation end products therapies as novel treatment for abdominal aortic aneurysm. Ann Surg 250: 416–423. 19652591 10.1097/SLA.0b013e3181b41a18 PMC2921961

[15] Kuzuya M, Iguchi A (2003) Role of matrix metalloproteinases in vascular remodeling. J Atheroscler Thromb 10: 275–282. 14718744 10.5551/jat.10.275

[16] Galis ZS, Khatri JJ (2002) Matrix metalloproteinases in vascular remodeling and atherogenesis: the good, the bad, and the ugly. Circ Res 90: 251–262. 11861412

[17] Qiu J, Xu J, Zheng Y, Wei Y, Zhu X, et al (2010) High-mobility group box 1 promotes metalloproteinase-9 upregulation through Toll-like receptor 4 after cerebral ischemia. Stroke 41: 2077–2082. 20671243 10.1161/STROKEAHA.110.590463 PMC3066477

[18] Li H, Sun B (2007) Toll-like receptor 4 in atherosclerosis. J Cell Mol Med 11: 88–95. 17367503 10.1111/j.1582-4934.2007.00011.x PMC4401222

[19] Michelsen KS, Wong MH, Shah PK, Zhang W, Yano J, et al (2004) Lack of Toll-like receptor 4 or myeloid differentiation factor 88 reduces atherosclerosis and alters plaque phenotype in mice deficient in apolipoprotein E. Proc Natl Acad Sci U S A. 101: 10679–10684. 10.1073/pnas.0403249101 PMC489994 15249654

[20] Ye X, Chopp M, Liu X, Zacharek A, Cui X, et al (2011) Niaspan reduces high-mobility group box 1/receptor for advanced glycation endproducts after stroke in type-1 diabetic rats. Neuroscience 190: 339–345. 21683770 10.1016/j.neuroscience.2011.06.004 PMC3260534

[21] Juvela S, Poussa K, Porras M (2001) Factors affecting formation and growth of intracranial aneurysms: a long-term follow-up study. Stroke 32: 485–491. 11157187 10.1161/01.str.32.2.485

[22] Onda H, Yoneyama T, Akagawa H, Kasuya H (2008) [Genetic dissection of intracranial aneurysm]. Brain Nerve 60: 1245–1260. 19069158

[23] Tada Y, Kitazato KT, Yagi K, Shimada K, Matsushita N, et al (2011) Statins promote the growth of experimentally induced cerebral aneurysms in estrogen-deficient rats. Stroke 42: 2286–2293. 21737796 10.1161/STROKEAHA.110.608034

[24] Guerreiro NE, Colli BO, Carlotti CG Jr, Chimelli L (2004) Experimental microaneurysms in rats: I. Model for induction. Surg Neurol 62: 406–412. 15518846 10.1016/j.surneu.2004.01.023

[25] Aoki T, Nishimura M, Kataoka H, Ishibashi R, Nozaki K, et al (2011) Complementary inhibition of cerebral aneurysm formation by eNOS and nNOS. Lab Invest 91: 619–626. 21321533 10.1038/labinvest.2010.204

[26] Calza L, Giardino L, Giuliani A, Aloe L, Levi-Montalcini R (2001) Nerve growth factor control of neuronal expression of angiogenetic and vasoactive factors. Proc Natl Acad Sci U S A 98: 4160–4165. 11259645 10.1073/pnas.051626998 PMC31196

[27] Chen J, Zhang ZG, Li Y, Wang Y, Wang L, et al (2003) Statins induce angiogenesis, neurogenesis, and synaptogenesis after stroke. Ann Neurol 53: 743–751. 12783420 10.1002/ana.10555

[28] Chen J, Li Y, Wang L, Zhang Z, Lu D, et al (2001) Therapeutic benefit of intravenous administration of bone marrow stromal cells after cerebral ischemia in rats. Stroke 32: 1005–1011. 11283404 10.1161/01.str.32.4.1005

[29] Aoki T, Kataoka H, Ishibashi R, Nozaki K, Hashimoto N (2008) Simvastatin suppresses the progression of experimentally induced cerebral aneurysms in rats. Stroke 39: 1276–1285. 18309148 10.1161/STROKEAHA.107.503086

[30] Creager MA, Luscher TF, Cosentino F, Beckman JA (2003) Diabetes and vascular disease: pathophysiology, clinical consequences, and medical therapy: Part I. Circulation. 108: 1527–1532. 10.1161/01.CIR.0000091257.27563.32 14504252

[31] Luscher TF, Creager MA, Beckman JA, Cosentino F (2003) Diabetes and vascular disease: pathophysiology, clinical consequences, and medical therapy: Part II. Circulation 108: 1655–1661. 14517152 10.1161/01.CIR.0000089189.70578.E2

[32] Thompson RW, Geraghty PJ, Lee JK (2002) Abdominal aortic aneurysms: basic mechanisms and clinical implications. Curr Probl Surg 39: 110–230. 11884965 10.1067/msg.2002.121421

[33] Lederle FA, Johnson GR, Wilson SE, Chute EP, Littooy FN, et al (1997) Prevalence and associations of abdominal aortic aneurysm detected through screening. Aneurysm Detection and Management (ADAM) Veterans Affairs Cooperative Study Group. Ann Intern Med 126: 441–449. 9072929 10.7326/0003-4819-126-6-199703150-00004

[34] Golledge J, Muller J, Daugherty A, Norman P (2006) Abdominal aortic aneurysm: pathogenesis and implications for management. Arterioscler Thromb Vasc Biol 26: 2605–2613. 16973970 10.1161/01.ATV.0000245819.32762.cb

[35] Lagares A, Gomez PA, Lobato RD, Alen JF, Campollo J, et al (1999) Cerebral aneurysm rupture after r-TPA thrombolysis for acute myocardial infarction. Surg Neurol 52: 623–626. 10660031 10.1016/s0090-3019(99)00147-0

[36] Festa A, D'Agostino R Jr, Mykkanen L, Tracy RP, Zaccaro DJ, et al (1999) Relative contribution of insulin and its precursors to fibrinogen and PAI-1 in a large population with different states of glucose tolerance. The Insulin Resistance Atherosclerosis Study (IRAS). Arterioscler Thromb Vasc Biol 19: 562–568. 10073958 10.1161/01.atv.19.3.562

[37] Kittaka M, Wang L, Sun N, Schreiber SS, Seeds NW, et al (1996) Brain capillary tissue plasminogen activator in a diabetes stroke model. Stroke 27: 712–719. 8614937 10.1161/01.str.27.4.712

[38] Dua MM, Miyama N, Azuma J, Schultz GM, Sho M, et al (2010) Hyperglycemia modulates plasminogen activator inhibitor-1 expression and aortic diameter in experimental aortic aneurysm disease. Surgery 148: 429–435. 20561659 10.1016/j.surg.2010.05.014 PMC2905480

[39] Astrand H, Ryden-Ahlgren A, Sundkvist G, Sandgren T, Lanne T (2007) Reduced aortic wall stress in diabetes mellitus. Eur J Vasc Endovasc Surg 33: 592–598. 17164093 10.1016/j.ejvs.2006.11.011

[40] Asari S, Ohmoto T (1993) Natural history and risk factors of unruptured cerebral aneurysms. Clin Neurol Neurosurg 95: 205–214. 8242963 10.1016/0303-8467(93)90125-z

[41] Winn HR, Almaani WS, Berga SL, Jane JA, Richardson AE (1983) The long-term outcome in patients with multiple aneurysms. Incidence of late hemorrhage and implications for treatment of incidental aneurysms. J Neurosurg 59: 642–651. 6886785 10.3171/jns.1983.59.4.0642

[42] Epstein M, Sowers JR (1992) Diabetes mellitus and hypertension. Hypertension 19: 403–418. 1568757 10.1161/01.hyp.19.5.403

[43] Kannel WB, McGee DL (1979) Diabetes and cardiovascular disease. The Framingham study. JAMA 241: 2035–2038. 430798 10.1001/jama.241.19.2035

[44] Taylor CL, Yuan Z, Selman WR, Ratcheson RA, Rimm AA (1995) Cerebral arterial aneurysm formation and rupture in 20,767 elderly patients: hypertension and other risk factors. J Neurosurg 83: 812–819. 7472548 10.3171/jns.1995.83.5.0812

[45] Kwak R, Mizoi K, Katakura R, Suzuki J (1979) The correlation between hypertension in past history and the incidence of cerebral aneurysms. Tohoku J Exp Med 128: 267–271. 494249 10.1620/tjem.128.267

[46] Fan X, Lo EH, Wang X (2013) Effects of minocycline plus tissue plasminogen activator combination therapy after focal embolic stroke in type 1 diabetic rats. Stroke 44: 745–752. 23422086 10.1161/STROKEAHA.111.000309 PMC3632085

[47] Ning R, Chopp M, Yan T, Zacharek A, Zhang C, et al (2012) Tissue plasminogen activator treatment of stroke in type-1 diabetes rats. Neuroscience 222: 326–332. 22820263 10.1016/j.neuroscience.2012.07.018 PMC3474540

[48] Yamagishi S, Nakamura K, Imaizumi T (2005) Advanced glycation end products (AGEs) and diabetic vascular complications. Curr Diabetes Rev 1: 93–106. 18220586 10.2174/1573399052952631

[49] Schmidt AM, Stern DM (2000) RAGE: a new target for the prevention and treatment of the vascular and inflammatory complications of diabetes. Trends Endocrinol Metab 11: 368–375. 11042467 10.1016/s1043-2760(00)00311-8

[50] Schmidt AM, Yan SD, Yan SF, Stern DM (2001) The multiligand receptor RAGE as a progression factor amplifying immune and inflammatory responses. J Clin Invest 108: 949–955. 11581294 10.1172/JCI14002 PMC200958

[51] Lee AJ, Fowkes FG, Carson MN, Leng GC, Allan PL (1997) Smoking, atherosclerosis and risk of abdominal aortic aneurysm. Eur Heart J 18: 671–676. 9129900 10.1093/oxfordjournals.eurheartj.a015314

[52] Huttunen HJ, Fages C, Rauvala H (1999) Receptor for advanced glycation end products (RAGE)-mediated neurite outgrowth and activation of NF-kappaB require the cytoplasmic domain of the receptor but different downstream signaling pathways. J Biol Chem 274: 19919–19924. 10391939 10.1074/jbc.274.28.19919

[53] Takeshita H, Yoshizaki T, Miller WE, Sato H, Furukawa M, et al (1999) Matrix metalloproteinase 9 expression is induced by Epstein-Barr virus latent membrane protein 1 C-terminal activation regions 1 and 2. J Virol 73: 5548–5555. 10364303 10.1128/jvi.73.7.5548-5555.1999 PMC112612

[54] Aoki T, Nishimura M, Ishibashi R, Kataoka H, Takagi Y, et al (2010) Toll-like receptor 4 expression during cerebral aneurysm formation. Laboratory investigation. J Neurosurg 113: 851–858. 19852543 10.3171/2009.9.JNS09329

[55] Grenier D, Grignon L (2006) Response of human macrophage-like cells to stimulation by Fusobacterium nucleatum ssp. nucleatum lipopolysaccharide. Oral Microbiol Immunol 21: 190–196. 16626377 10.1111/j.1399-302X.2006.00278.x

[56] Kanematsu Y, Kanematsu M, Kurihara C, Tada Y, Tsou TL, et al (2011) Critical roles of macrophages in the formation of intracranial aneurysm. Stroke 42: 173–178. 21106959 10.1161/STROKEAHA.110.590976 PMC3021554

[57] Hodgkinson CP, Patel K, Ye S (2008) Functional Toll-like receptor 4 mutations modulate the response to fibrinogen. Thromb Haemost 100: 301–307. 18690351

[58] Naduk-Kik J, Hrabec E (2008) [The role of matrix metalloproteinases in the pathogenesis of diabetes mellitus and progression of diabetes retinopathy]. Postepy Hig Med Dosw (Online) 62: 442–450. 18772849

[59] Dollery CM, McEwan JR, Henney AM (1995) Matrix metalloproteinases and cardiovascular disease. Circ Res 77: 863–868. 7554139 10.1161/01.res.77.5.863

[60] Longo GM, Xiong W, Greiner TC, Zhao Y, Fiotti N, et al (2002) Matrix metalloproteinases 2 and 9 work in concert to produce aortic aneurysms. J Clin Invest 110: 625–632. 12208863 10.1172/JCI15334 PMC151106

[61] McMillan WD, Tamarina NA, Cipollone M, Johnson DA, Parker MA, et al (1997) Size matters: the relationship between MMP-9 expression and aortic diameter. Circulation 96: 2228–2232. 9337194 10.1161/01.cir.96.7.2228

[62] Prall AK, Longo GM, Mayhan WG, Waltke EA, Fleckten B, et al (2002) Doxycycline in patients with abdominal aortic aneurysms and in mice: comparison of serum levels and effect on aneurysm growth in mice. J Vasc Surg 35: 923–929. 12021708 10.1067/mva.2002.123757

[63] Lindeman JH, Abdul-Hussien H, van Bockel JH, Wolterbeek R, Kleemann R (2009) Clinical trial of doxycycline for matrix metalloproteinase-9 inhibition in patients with an abdominal aneurysm: doxycycline selectively depletes aortic wall neutrophils and cytotoxic T cells. Circulation 119: 2209–2216. 19364980 10.1161/CIRCULATIONAHA.108.806505

